# Use of Blood Lipid Indicators as a Screening Tool of Insulin Resistance among Individuals in Low-Income Country Sides of China: A Multiethnic Region Study

**DOI:** 10.1155/2019/3592620

**Published:** 2019-10-07

**Authors:** Yi-Zhong Yan, Jia-Ning Fan, Jia-Ming Liu, Yun-hua Hu, Jiao-Long Ma, Jia He, Heng Guo, Xiang-hui Zhang, Xin-ping Wang, Shu-gang Li, Shu-Xia Guo

**Affiliations:** ^1^Department of Public Health, Shihezi University School of Medicine, Shihezi Xinjiang 832002, China; ^2^Department of Pathology and Key Laboratory of Xinjiang Endemic and Ethnic Diseases (Ministry of Education), Shihezi University School of Medicine, Shihezi Xinjiang 832002, China

## Abstract

**Objective:**

This study is aimed at evaluating the diagnostic value of blood lipid indicators (BLIs) for insulin resistance (IR) among major ethnic groups in Xinjiang, China, to identify the most valuable indicators and appropriate cut-off points for each ethnic group and to lay the foundation for the early detection, diagnosis, and treatment of metabolic diseases in remote rural areas.

**Methods:**

Overall, 418 Uygurs, 331 Kazakhs, and 220 Hans were randomly included in our study. The homeostasis model assessment was the gold standard for identifying IR. The receiver operating characteristic (ROC) curve was used to evaluate the diagnostic value, and the nomogram was utilized to analyze the predictive value. The size of the area under the curve (AUC) reflected the accuracy of screening and prediction.

**Results:**

Differences in races were observed in terms of IR and BLIs, and the Kazakhs had the highest IR level at 5.27 mmol/L. The correlation between IR and BLIs differed among the three races. For the Kazakhs and Hans, all BLIs, except total cholesterol (TC), were correlated to IR. However, for the Uygurs, only the triglyceride (TG) level, TG/high-density lipoprotein cholesterol (HDL-C) ratio, and TC/HDL-C ratio were associated with IR. After further adjustment of confounding factors, these indicators were still correlated to IR. BLIs that independently correlated to IR in the three nationalities had a certain diagnostic value for IR. In terms of the AUC size, the TG level was the highest in Uygurs, the TG/HDL-C ratio was the highest for Kazakhs and Hans, and the corresponding best cut-off points for IR were 1.515, 1.230, and 1.495 mmol/L, respectively. In addition, for each race, when the indicators with a certain diagnostic value were combined, the diagnostic value for IR was higher.

**Conclusion:**

BLIs had a certain diagnostic value for IR and could be used as a screening tool for IR among Uygurs, Kazakhs, and Hans in Xinjiang. These findings are extremely important for the prevention and treatment of IR and metabolic diseases in remote rural areas.

## 1. Introduction

IR is the collective physiological and pathological bases of metabolic diseases, such as abdominal obesity, hypertension, diabetes, and dyslipidemia, and it is considered the central link of the development of such diseases [1, 2]. Moreover, IR and insufficient insulin secretion are the main factors associated with the development of type 2 diabetes mellitus (T2DM) and are regarded as the important clinical markers for the early diagnosis and late follow-up of such condition [3]. And quite a few studies have shown that dyslipidemia was an early manifestation of IR [4], and some scholars believed that hypertriglyceridemia was indeed a representation of IR [5]. Simultaneously, TG and HDL-C levels were found to be negatively and positively, respectively, correlated to insulin sensitivity index [6, 7]. Consequently, the focus of studies has gradually shifted from the initial view that glycometabolism disorder was the primary manifestation of IR and T2DM to the notion that abnormal blood lipid was the first manifestation and core link to their onset [8, 9].

To date, IR has been identified as a potential mechanism of numerous chronic diseases. However, its role in predicting the risk of a disease and establishing effective treatment strategies has been limited due to the lack of practical quantitative and universal diagnostic methods. Previously, the hyperinsulinemic clamp technique was used as a golden index for the evaluation of IR [10]. However, it was challenging to use in patients due to its complex nature and high cost. At present, the homeostasis model assessment as an index of insulin resistance (HOMA-IR) is the recognized diagnostic criterion [11]; however, this tool requires the identification of fasting insulin in the serum (FINS), and standardized methods for it in laboratories are limited. In addition, FINS is susceptible to the influence of islet *β* cell function and hypoglycemic drugs. All the disadvantages will make the implementation of clinical and epidemiological research challenging, and IR will be more difficult to be identified in natural population. Instead, BLIs can be easily identified in regular physical examinations and epidemiological surveys, and their close correlation to IR also provided opportunities and possibilities for the early detection and screening of IR. A superior predictive value has been attributed to lipoprotein ratios, such as TC/HDL-C and LDL-C/HDL-C ratios [7]. In particular, the TG/HDL-C ratio has emerged as a marker of IR [12] in nondiabetic individuals as well as a good predictor of metabolic syndrome (MS) and cardiovascular disease (CVD) [13].

In addition, numerous studies have presented the importance of ethnic characteristics on adequate cut-off points for the identification of individuals at the risk of IR. China is a multiethnic country, and a high number of studies have reported about the Han population in central China [14, 15]; only few have assessed the Uygur, Kazakh, and Han people living in the remote rural areas of China. At the same time, we know that common metabolic diseases are significantly correlated to ethnic customs, living environment, and lifestyle habits. Analyzing the relationship between BLIs and IR in different populations and corresponding diagnostic thresholds is of great importance for effective and targeted disease control and improvement of the health of the nation. Moreover, simple and inexpensive means, such as screening for IR using BLIs, were more in demand in areas with relatively backward economic and medical conditions, which include areas in Xinjiang, China. In the present study, the Uygur, Kazakh, and Han populations in the Xinjiang region of China were included, and the relationship between BLIs and IR was analyzed, the ROC curve was obtained, and the threshold of IR screening for each ethnic group was identified, which provided the basis for the establishment and improvement of IR screening and diagnostic strategies in Xinjiang.

## 2. Participants and Methods

### 2.1. Subjects

Our survey was conducted from 2009 to 2013 in the five regions of Xinjiang, including Ili Kazak Autonomous Prefecture, Kashgar Prefecture, Shihezi, Tarbagatay Prefecture, and Changji Hui Autonomous Prefecture. This survey collected information about chronic disease (including metabolic syndrome, hypertension, diabetes mellitus, dyslipidemia, and cardiovascular disease) from residents (≥18 years old and having lived locally for more than 10 years). On this basis, we randomly selected subjects for experimental detection of this study. The formula for estimating the sample size is as follows:

(1) $$\begin{array}{c} n={\left(\frac{z_{\alpha }}{\delta }\right)}^{2}\left(1-p\right)p. \end{array}$$

In the formula, *n* is the sample size. The common values of *Z*_*α*_ are *Z*_0.05/2_ = 1.96 or *Z*_0.01/2_ = 2.58; in this study, 1.96 was chosen; *δ* is the allowable error, generally 0.05-0.1, and our study taken 0.1; *P* is the sensitivity or specificity of the screening methods to be evaluated. The sample size of the case group is usually estimated by sensitivity, and that of the control group is estimated by specificity. One study showed that the sensitivity and specificity of BLIs in screening IR in Chinese Han population were 70.1% and 66.1% [7]. Based on the above formulas, 167 samples were estimated in this study. Considering the influence of blood samples, reagents, and experimental operations, the estimated sample size was expanded. Finally, the sample size (418, 331, and 220 cases for Uygurs, Kazakhs, and Hans, respectively) was determined according to the actual results of detection.

### 2.2. Gold Standard of IR

The homeostasis model assessment was applied to assess the status of IR (HOMA-IR) and was defined as follows: [fasting insulin (mU/L) × fasting glucose (mM)]/22.5. The Chinese Diabetes Society states that IR can be estimated using this formula in epidemiological or clinical studies, and the upper quartile of the subjects was the split point [16]. The boundaries in this study were 3.28, 5.75, and 2.82 for Uygurs, Kazakhs, and Hans, respectively.

### 2.3. Laboratory Tests

TC, TG, LDL-C, HDL-C, and fasting glucose levels were assessed by a biochemical autoanalyzer (Olympus AU 2700, Olympus Diagnostics, Hamburg, Germany) in a clinical laboratory. FINS was determined by radioimmunoassay with a kit purchased from Beijing Atomic Tech Co. Ltd. (Beijing, China).

### 2.4. Statistical Analysis

All of the analyses were performed with SPSS (version 25.0) and statistical software packages R (https://www.r-project.org/, The R Foundation) and Empower Stats (http://www.empowerstats.com, X&Y Solutions, Inc., Boston, MA). Comparisons of the general clinical data between the two groups (IR and non-IR groups) were performed using Student's *t*-test for continuous variables (e.g., age, TG, and TC) and chi-square tests for categorical variables (e.g., sex). The multivariate logistic regression analysis was carried out to identify the independent risk factors of IR through stepwise selection. A nomogram was established based on the results of the multivariate analysis. The diagnostic value of BLIs was measured using an area under the ROC curve (AUC). In addition, an ROC curve was obtained to determine the optimal threshold of the nomogram. A two-sided *P* value < 0.05 was considered statistically significant.

## 3. Results

### 3.1. Description of the Basic Information

A total of 969 participants were included in the study, of which 418 (43.1%) were Uygurs, 331 (34.2%) were Kazakhs, and 220 (22.7%) were Hans. First, we compared the IR and BLIs among the three nationalities, and results showed that such indicators significantly differed in terms of race (*P* < 0.05 for each comparison) (Table 1). Then, each ethnic group was divided into two groups (non-IR and IR groups) in accordance with the high quartile of IR. Based on the statistical analysis of the clinical characteristics of the two groups in each of these three nationalities, age and sex did not significantly differ (*P* > 0.05 for each comparison). In addition, BMI, TG level, TG/HDL-C ratio, and TC/HDL-C ratio significantly differed between the two groups for Uygurs and Kazakhs, and the TG level, HDL-C level, TG/HDL-C ratio, TC/HDL-C ratio, LDL-C/HDL-C ratio, and 1 mmol/HDL-C level were obviously different for Hans (*P* < 0.05 for each comparison) (Table 2).

### 3.2. Multivariate Logistic Regression Analysis

To eliminate the confounding effects of other factors on the relationship between BLIs and IR, we used multivariate logistic regression analysis to adjust such factors. Results showed that the BMI, TG level, TG/HDL-C ratio, and TC/HDL-C ratio of the Uygurs were included using the regression equation, and the TG level, HDL-C level, TG/HDL-C ratio, TC/HDL-C ratio, LDL-C/HDL-C ratio, and 1/HDL-C level of the Kazakh still differed significantly after adjusting for other factors. The findings observed in Hans were similar to those in Kazakhs (Table 3).

### 3.3. Use of the Nomogram

**Figure 1:**
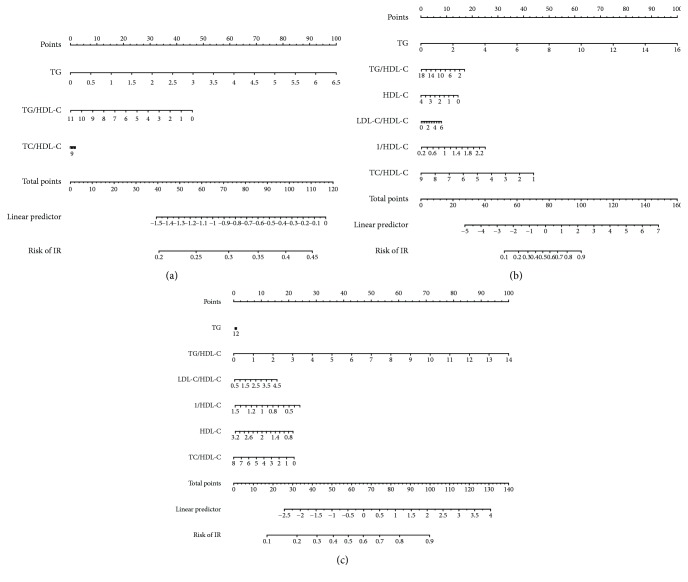
Nomogram to estimate the risk of IR. (a) Nomogram for Uygurs. (b) Nomogram for Kazakhs. (c) Nomogram for Hans. To use the nomogram, find the position of each variable on the corresponding axis, draw an upward vertical line to the point axis to get the relevant number of points, add the points from all of the variables, and draw a line from the total point axis to determine the IR probabilities at the lower line of the nomogram. TG = triglyceride; TC = total cholesterol; HDL-C = high-density lipoprotein cholesterol; LDL-C = low-density lipoprotein cholesterol.

For example, in Uygurs, if the TG level was 4 mmol/L, an upward vertical line can be drawn from the TG axis to the point bar to obtain the point of 25. Other indicators can also be manipulated such as this, and we assumed that the TG/HDL-C ratio of 6 had a score of 10 points, the HDL-C level of 1 mmol/L had 10 points, the LDL-C/HDL-C ratio of 4 had 5 points, the 1/HDL-C level of 0.8 had 6 points, and the TC/HDL-C ratio of 6 had 15 points. Then, the total score was 71 (25 + 10 + 10 + 5 + 6 + 15). Finally, the risk of IR was about 20% when we marked a straight line from the total points to the risk of the IR axis (Figure 1).

### 3.4. Screening Value and Cut-off Point Analysis

**Figure 2:**
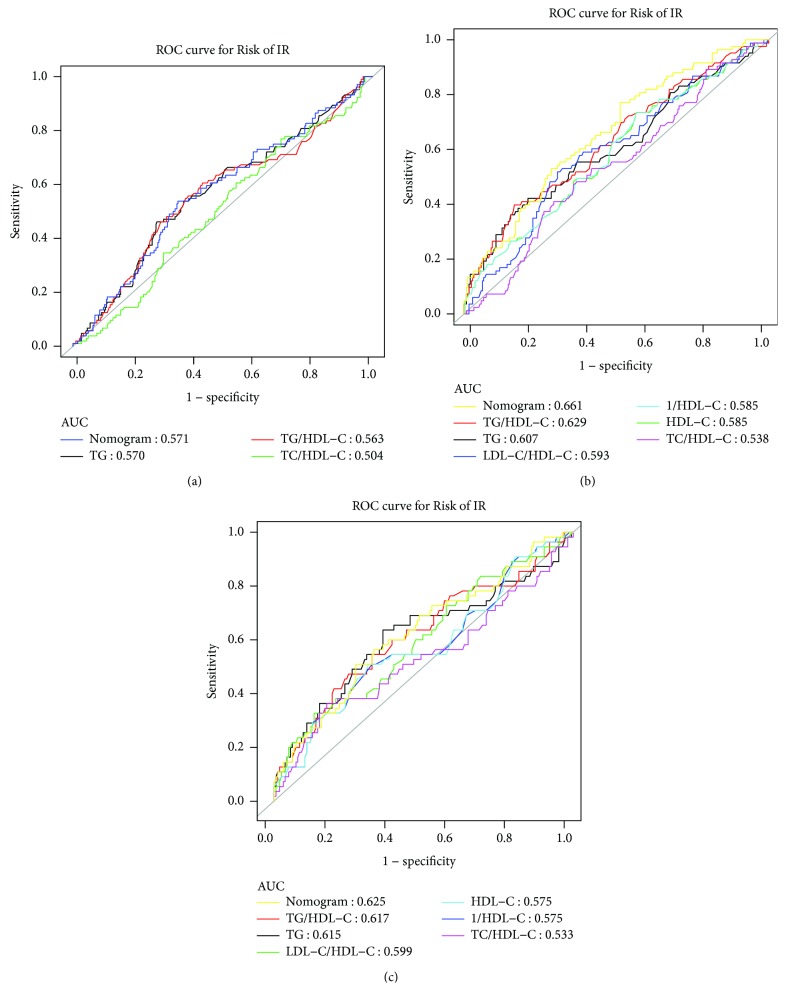
ROC curve of nomogram and other models to diagnose IR. (a) ROC curve for Uygurs. (b) ROC curve for Kazakhs. (c) ROC curve for Hans. TG = triglyceride; TC = total cholesterol; HDL-C = high-density lipoprotein cholesterol; LDL-C = low-density lipoprotein cholesterol.

In Uygurs, the AUC of the nomogram for the prediction of IR prediction was 0.571, and the optimal cut-off value was -1.123. Among the single indicators, the TG level had the largest AUC at 0.570, and the optimal cut-off value was 1.515. The AUC of the nomogram for Kazakhs was 0.661, and the optimal cut-off value was -1.321. Moreover, among the single indicators, the TG/HDL-C ratio had the largest AUC at 0.629, and the optimal cut-off was 1.230. For Hans, the AUC of the nomogram was 0.625, and the optimal cut-off value was -1.074. The TG/HDL-C ratio had the largest AUC at 0.617 among the single indicators, and the optimal cut-off value was 1.495 (Table 4, Figure 2).

## 4. Discussion

In view of the global epidemic of metabolic diseases in recent years, including DM, MS, or CVD, it was of great clinical and preventive significance to develop a simple and inexpensive screening tool to help clinicians quickly identify individuals who are at risk. The pathogenesis of these diseases has been linked to IR, which was found to be associated with dyslipidemia, including low HDL-C level, high TG level, and postprandial lipid level [17–19], and all these atherosclerotic lipid abnormalities preceded other symptoms of diabetes, such as elevated blood sugar level, and another obvious advantage was that they were easy to detect. The current research systematically analyzed the relationship between various BLIs and IR among three major ethnic groups in remote areas of China. In relation to this, exploratory analysis was made on the predictive and diagnostic value of these indices for IR and the appropriate cut-off points, which will lay a foundation for the screening and diagnosis of metabolic diseases, such as IR and diabetes.

Significant racial differences were observed in terms of IR and various related indicators of blood lipid. In this study, the homeostasis model assessment method was used to calculate the HOMA-IR [16]. Results showed significant differences in IR levels among the Uygurs, Kazakhs, and Hans, and the Kazakhs had the highest IR level at 5.27 mmol/L. Previous studies have found that the prevalence of dyslipidemia, DM, and MS in Kazakhs was higher than that in Uygurs and Hans [20], indicating that the state of IR and metabolic diseases was accordant among the three races, and this result coincided with the view that IR was the pathological basis of metabolic diseases. In addition, the levels of BLIs were also distinct, and all these phenomena indicated that indexes and criteria should be selected and formulated according to different crowds, which was conducive in improving the sensitivity and specificity of the screening tool [15, 21].

To scientifically and accurately select the screening tools for IR, correlation analysis was carried out in our study. First, according to the HOMA-IR, the participants of all nationalities were divided into the IR and non-IR groups for univariate analysis. We found that the relevance between IR and BLIs among the different nationalities was different, but the TG level and TG/HDL-C ratio were correlated to IR in all three races, which was consistent with the conclusion of other studies showing that the TG/HDL-C ratio can be used as an alternative index to diagnose IR [7, 22]. Second, multivariate logistic regression analysis was used to determine the independence of each index. After adjusting for confounding factors, the TG level and TG/HDL-C ratio of the three multitudes were still correlated to IR, and these independent indexes may be significant for the diagnosis of IR [23].

The screening value of each lipid index and the optimal cut-off point varied in different races. According to the AUC of the ROC curve, the index with an AUC > 0.5 had a certain diagnostic value [24]. The indexes included in the diagnostic test in our study had a diagnostic value for IR. Among them, the TG level of Uygurs as well as the TG/HDL-C ratio of Kazakhs and Hans was marginally stronger than other respective indices as markers of IR. In addition, the corresponding optimal cut-off points also differed (1.515, 1.230, and 1.495, respectively). All these results were lower than those for Americans (3.5 [25] and 3.0 [26]), which is probably due to the fact that the basic TG/HDL-C ratios of the participants were not similar. Meanwhile, compared with the Han population in other areas, the cut-off point of TG/HDL-C in our study was somewhat higher [27], and this result indicated regional differences in terms of thresholds, which must be considered when the diagnostic criteria were established, particularly in areas with large economic and cultural disparity. Moreover, this study evaluated the value of the combined screening of the indicators for IR, and results showed that the AUC after the union of each ethnic group was higher than that of the individual index. This result showed that when conditions permit, we can combine lipid indices to diagnose IR, which may improve the screening effect to a certain extent.

This study had several limitations. First, this study included three main ethnic groups in the remote northwest of China. Although the sample was abundant, the sample size of each ethnic group was relatively small. This may lead to the exclusion of some key indicators in the diagnostic tests. For example, in Uygurs, only three indicators were eventually included. Second, although more variables have been included when we analyzed the confounding factors, some sociological factors have not been considered, which may have an impact on the judgment of the independence of the relevant indicators. Therefore, the screening value of the BLIs for IR must be further analyzed after expanding the samples and variables to obtain more scientific and accurate diagnostic indicators and the best cut-off point.

In summary, a series of metabolic diseases have become a major public health problem, and early detection, diagnosis, and treatment are the primary tasks that prevent and treat these diseases. This study is aimed at analyzing the screening value of BLIs for IR to obtain the best index and cut-off point with national suitability. After a multiangle analysis, it was concluded that the screening value after union of BLIs for IR was the highest. In terms of independent BLIs, TG had the most prominent screening value for IR in Uygurs, while the value of TG/HDL-C ratio was the highest for both Kazakhs and Hans; at the same time, racial differences also showed up at the best cut-off point. This kind of research was important for governments to establish and carry out simple, inexpensive, efficient, and feasible screening tools. In the future, we can further evaluate the diagnostic value of BLIs for IR, T2DM, MS, and CVD in wider remote rural areas to improve the survival rate and quality of life of patients.

## Figures and Tables

**Table 1:** Comparison of BLIs among three nationalities.

Nation	Uygurs (*n* = 418)	Kazakhs (*n* = 331)	Hans (*n* = 220)	*F* value	*P* value
HOMA-IR	3.21 ± 0.22	5.27 ± 0.45	2.98 ± 0.58	10.733	0.000
TG (mmol/L)	1.41 ± 0.92	1.31 ± 0.24	1.77 ± 0.28	11.939	0.000
TC (mmol/L)	4.51 ± 1.13	4.47 ± 1.04	4.48 ± 1.08	5.385	0.005
HDL-C (mmol/L)	1.18 ± 0.28	1.38 ± 0.39	1.43 ± 0.35	53.270	0.000
LDL-C (mmol/L)	2.50 ± 0.73	2.32 ± 0.74	2.79 ± 0.77	26.699	0.000
TG/HDL-C	1.38 ± 0.29	1.09 ± 0.48	1.41 ± 0.46	5.065	0.006
TC/HDL-C	4.03 ± 1.35	3.38 ± 0.95	3.46 ± 0.94	36.071	0.000
LDL-C/HDL-C	2.23 ± 0.82	1.78 ± 0.69	2.04 ± 0.66	34.987	0.000
1/HDL-C (1/mmol/L)	0.90 ± 0.24	0.78 ± 0.21	0.74 ± 0.18	51.615	0.000

Notes: HOMA-IR = homeostasis model assessment of insulin resistance; TG = triglyceride; TC = total cholesterol; HDL-C = high-density lipoprotein cholesterol; LDL-C = low-density lipoprotein cholesterol.

**Table 2:** Comparison of general clinical data.

Index	Uygurs (*n* = 418)				Kazakhs (*n* = 331)				Hans (*n* = 220)			
	Non-IR	IR	*χ* ^2^/*t* value	*P* value	Non-IR	IR	*χ* ^2^/*t* value	*P* value	Non-IR	IR	*χ* ^2^/*t* value	*P* value
Sex (males/females)	144/170	54/50	1.152	0.283	104/144	42/41	1.895	0.169	79/86	19/36	2.969	0.085
Age (year)	41.71 ± 11.76	41.52 ± 13.34	0.144	0.885	45.73 ± 10.79	46.64 ± 11.77	0.363	0.716	49.31 ± 10.65	50.00 ± 11.25	-0.411	0.682
Weight (kg)	59.84 ± 12.18	62.49 ± 12.32	-1.924	0.055	67.59 ± 12.82	69.39 ± 13.68	-0.660	0.510	64.98 ± 10.41	67.11 ± 10.73	-1.305	0.193
Height (cm)	159.62 ± 8.21	160.10 ± 8.69	-0.511	0.609	163.27 ± 7.83	163.94 ± 8.36	-1.088	0.277	161.66 ± 8.00	161.45 ± 7.13	0.174	0.862
Waistline (cm)	85.39 ± 11.17	87.25 ± 12.22	-1.439	0.151	87.96 ± 11.02	89.82 ± 12.36	-1.289	0.198	86.57 ± 9.95	89.74 ± 9.75	-2.058	0.041
Hipline (cm)	95.60 ± 7.91	96.18 ± 9.09	-0.625	0.533	98.30 ± 7.15	98.64 ± 7.07	-0.375	0.708	96.69 ± 6.50	98.03 ± 7.18	-1.287	0.199
SBP (mmHg)	122.15 ± 19.08	124.05 ± 18.67	-0.883	0.378	139.69 ± 26.19	140.41 ± 25.73	-0.218	0.828	130.06 ± 23.42	131.93 ± 25.52	-0.501	0.617
DBP (mmHg)	78.16 ± 12.64	78.77 ± 11.91	-0.430	0.668	89.10 ± 14.59	88.34 ± 14.76	0.407	0.684	84.78 ± 14.06	84.49 ± 13.69	0.132	0.895
WHR	0.89 ± 0.07	0.91 ± 0.08	-1.672	0.095	0.89 ± 0.07	0.91 ± 0.08	-1.590	0.113	0.89 ± 0.07	0.92 ± 0.07	-1.770	0.078
BMI (kg/m^2^)	23.38 ± 3.78	24.27 ± 3.82	-2.105	0.036	25.31 ± 4.23	25.75 ± 4.34	-0.817	0.414	24.84 ± 3.29	25.75 ± 4.01	-1.684	0.094
TG (mmol/L)	1.36 ± 0.91	1.53 ± 0.95	-1.272	0.041	1.15 ± 0.67	1.81 ± 2.14	-2.747	0.007	1.59 ± 0.89	2.33 ± 1.96	-2.683	0.009
TC (mmol/L)	4.48 ± 1.17	4.61 ± 1.03	-1.007	0.314	4.45 ± 1.03	4.52 ± 1.06	-0.470	0.639	4.73 ± 0.98	4.86 ± 1.36	-0.658	0.512
HDL-C (mmol/L)	1.18 ± 0.28	1.16 ± 0.30	0.661	0.509	1.41 ± 0.40	1.29 ± 0.35	2.326	0.019	1.45 ± 0.35	1.35 ± 0.34	1.933	0.045
LDL-C (mmol/L)	2.48 ± 0.71	2.52 ± 0.82	-0.544	0.587	2.31 ± 0.72	2.38 ± 0.79	-0.750	0.454	2.76 ± 0.69	2.90 ± 0.94	-1.216	0.225
TG/HDL-C	1.33 ± 1.25	1.54 ± 1.41	-1.162	0.034	0.89 ± 0.68	1.67 ± 2.66	-2.642	0.010	1.20 ± 0.81	2.06 ± 1.45	-2.582	0.012
TC/HDL-C	4.22 ± 1.38	3.97 ± 1.34	-1.173	0.028	3.64 ± 1.00	3.29 ± 0.91	-2.952	0.003	3.76 ± 1.22	3.36 ± 0.80	-2.273	0.026
LDL-C/HDL-C	2.21 ± 0.81	2.31 ± 0.87	-1.005	0.315	1.73 ± 0.69	1.92 ± 0.66	-2.214	0.027	1.98 ± 0.60	2.24 ± 0.76	-2.378	0.020
1/HDL-C (1/mmol/L)	0.89 ± 0.23	0.92 ± 0.27	-1.032	0.303	0.76 ± 0.21	0.83 ± 0.22	-2.491	0.013	0.72 ± 0.16	0.79 ± 0.21	-2.093	0.040

Notes: SBP = systolic blood pressure; DBP = diastolic blood pressure; WHR = waist-to-hip ratio; BMI = body mass index; TG = triglyceride; TC = total cholesterol; HDL-C = high-density lipoprotein cholesterol; LDL-C = low-density lipoprotein cholesterol.

**Table 3:** Multivariate logistic regression analysis.

Variables	Uygurs			Kazakhs			Hans		
	*OR*	*95% CI for OR*	*Pvalue*	*OR*	*95% CI for OR*	*Pvalue*	*OR*	*95% CI for OR*	*Pvalue*
Waistline (cm)	—	—	—	—	—	—	2.259	(0.807, 4.962)	0.028
BMI (kg/m^2^)	2.025	(0.967, 4.087)	0.019	—	—	—	—	—	—
TG (mmol/L)	3.698	(1.265, 6.279)	0.000	2.215	(0.968, 5.525)	0.033	1.543	(1.180, 2.018)	0.002
HDL-C (mmol/L)	—	—	—	1.876	(0.879, 2.416)	0.029	2.189	(1.023, 7.567)	0.032
TG/HDL-C	4.645	(1.225, 9.208)	0.000	5.007	(0.935, 11.086)	0.000	4.529	(1.161, 9.012)	0.000
TC/HDL-C	2.357	(1.027, 6.107)	0.008	4.954	(1.345, 9.948)	0.000	5.412	(0.879, 10.246)	0.000
LDL-C/HDL-C	—	—	—	2.987	(0.919, 6.059)	0.011	1.854	(1.161, 2.862)	0.010
1/HDL-C (1/mmol/L)	—	—	—	1.957	(0.739, 4.238)	0.036	7.354	(1.360, 19.775)	0.021

Notes: BMI = body mass index; TG = triglyceride; TC = total cholesterol; HDL-C = high-density lipoprotein cholesterol; LDL-C = low-density lipoprotein cholesterol.

**Table 4:** Analysis of diagnostic tests and ROC curve.

Variables		AUC	95% CI for AUC	Cut-off	Spe	Sen
Uygurs	TG (mmol/L)	0.570	(0.505, 0.635)	1.515	0.720	0.462
	TG/HDL-C	0.563	(0.497, 0.629)	1.005	0.567	0.606
	TC/HDL-C	0.504	(0.441, 0.567)	3.175	0.306	0.769
	Nomogram	0.571	(0.506, 0.635)	-1.123	0.650	0.539

Kazakhs	TG (mmol/L)	0.607	(0.533, 0.681)	1.425	0.790	0.422
	HDL-C (mmol/L)	0.585	(0.514, 0.657)	1.435	0.436	0.735
	TG/HDL-C	0.629	(0.558, 0.701)	1.230	0.835	0.398
	TC/HDL-C	0.538	(0.468, 0.609)	2.885	0.706	0.410
	LDL-C/HDL-C	0.593	(0.522, 0.664)	1.905	0.694	0.518
	1/HDL-C (1/mmol/L)	0.585	(0.514, 0.657)	0.695	0.436	0.735
	Nomogram	0.661	(0.595, 0.728)	-1.321	0.488	0.771

Hans	TG (mmol/L)	0.615	(0.520, 0.710)	1.585	0.636	0.636
	TG/HDL-C	0.617	(0.526, 0.709)	1.495	0.752	0.473
	TC/HDL-C	0.533	(0.436, 0.630)	4.095	0.824	0.364
	LDL-C/HDL-C	0.599	(0.509, 0.689)	2.595	0.867	0.327
	1/HDL-C (1/mmol/L)	0.575	(0.483, 0.667)	0.775	0.673	0.509
	HDL-C (mmol/L)	0.575	(0.483, 0.667)	1.285	0.685	0.509
	Nomogram	0.625	(0.537, 0.713)	-1.074	0.727	0.582

Notes: TG = triglyceride; TC = total cholesterol; HDL-C = high-density lipoprotein cholesterol; LDL-C = low-density lipoprotein cholesterol; Sen = sensitivity; Spe = specificity.

## Data Availability

The data used to support the findings of this study are available from the corresponding authors upon request.

## Abbreviations

AUC: Area under the curve
BLIs: Blood lipid indicators
CI: Confidence interval
CVD: Cardiovascular disease
DBP: Diastolic blood pressure
FINS: Fasting insulin
HDL-C: High-density lipoprotein cholesterol
HOMA-IR: Homeostasis model assessment of insulin resistance
IR: Insulin resistance
LDL-C: Low-density lipoprotein cholesterol
MS: Metabolic syndrome
OR: Odds ratio
ROC: Receiver operating characteristic
SBP: Systolic blood pressure
TC: Total cholesterol
TG: Triglyceride
T2DM: Type 2 diabetes mellitus
WC: Waist circumference.
